# Racial disparities in caesarean delivery among nulliparous women that delivered at term: cross-sectional decomposition analysis of Nebraska birth records from 2005-2014

**DOI:** 10.1186/s12884-022-04666-3

**Published:** 2022-04-15

**Authors:** Corrine Hanson, Kaeli Samson, Ann L. Anderson-Berry, Rebecca A. Slotkowski, Dejun Su

**Affiliations:** 1 College of Allied Health Professions, Medical Nutrition Education, University of Nebraska Medical Center, Omaha, NE 68198-4045 USA; 2 College of Public Health, University of Nebraska Medical Center, Omaha, NE 68198-4340 USA; 3 Pediatrics, University of Nebraska Medical Center, Omaha, NE 68198-1205 USA

**Keywords:** Caesarean section, Racial disparities, Obesity, Maternal morbidity, Public health

## Abstract

**Background:**

Previous studies suggest higher rates of caesarean section among women who identify as racial/ethnic minorities. The objective of this study was to understand factors contributing to differences in caesarean rates across racial and ethnic groups.

**Methods:**

Data was collected from 2005 to 2014 Nebraska birth records on nulliparous, singleton births occurring on or after 37 weeks gestation (*n* = 87,908). Risk ratios (RR) and 95% confidence intervals (CI) for caesarean were calculated for different racial and ethnic categories, adjusting for maternal age, marital status, county of residence, education, insurance status, pre-pregnancy BMI, and smoking status. Fairlie decomposition technique was utilized to quantify the contribution of individual variables to the observed differences in caesarean.

**Results:**

In the adjusted analysis, relative to non-Hispanic (NH) White race, both Asian-NH (RR 1.21, 95% CI 1.14, 1.28) and Black-NH races (RR 1.13, 95% CI 1.08, 1.19) were associated with a significantly higher risk for caesarean. The decomposition analysis showed that among the variables assessed, maternal age, education, and pre-pregnancy BMI contributed the most to the observed differences in caesarean rates across racial/ethnic groups.

**Conclusion:**

This analysis quantified the effect of social and demographic factors on racial differences in caesarean delivery, which may guide public health interventions aimed towards reducing racial disparities in caesarean rates. Interventions targeted towards modifying maternal characteristics, such as reducing pre-pregnancy BMI or increasing maternal education, may narrow the gap in caesarean rates across racial and ethnic groups. Future studies should determine the contribution of physician characteristics, hospital characteristics, and structural determinants of health towards racial disparities in caesarean rates.

## Background

Access to medically indicated caesarean sections is an essential strategy for reducing maternal and infant mortality rates worldwide [1]. However, overuse of medically unnecessary caesarean sections is associated with excess maternal-child morbidity [1–3]. Additionally, primary caesareans contribute to the high prevalence of repeat caesareans [4] and impose an economic burden on the health care system [5, 6]. Despite national [7] and international [1] efforts to prioritize the reduction of medically unnecessary caesarean sections, caesareans rates in the United States have remained stable over the last decade [8, 9]. In 2019 the national caesarean rate for nulliparous low-risk (term, singleton, and vertex presentation) births was 25.6% [8], well above the target rate of 24.7% set by Heathy People 2020 [7].

Women who identify as racial or ethnic minorities experience disproportionally higher rates of caesarean in the United States [8], even when controlling for demographic, behavioral, medical, and institutional level factors [10–13]. Various hypotheses have been proposed to explain these differences, including biased or discriminatory variation in clinical decision making about labor management [14, 15], maternal preferences and risk tolerance [16], and unpredictability of labor patterns among subgroups of women [17]. However, detailed analysis of factors contributing to racial/ethnic disparities in caesarean section rates remains largely unexplored. Underlying racial and ethnic differences in caesarean use could be influenced by a host of factors including but not limited to demographics and health insurance coverage. Identifying these factors and assessing their relative importance is critical for the development and prioritization of interventions specifically tailored to reduce racial and ethnic disparities in caesarean use.

We undertook this study to identify risk factors for caesarean by maternal race/ethnicity using birth records provided by the Nebraska Department of Health and Human Services (DHHS). This was completed through a Fairlie decomposition analysis of identified explanatory factors to rank their importance based on the proportion of the observed racial/ethnic difference in caesarean rate accounted for by each factor. The relative contribution of specific factors that explain differences in caesarean rate provides a more in-depth understanding of disparities, which can inform future policy and interventional decision-making.

## Methods

This study was a cross-sectional analysis of data collected from birth records reported to the vital records electronic registration system at the Nebraska DHHS for the period from 2005 to 2014. This information is collected concurrently with the application for a certificate of live birth in the State of Nebraska. IRB approval was not required as all data was de-identified and safeguarded by Nebraska DHHS. Inclusion criteria were nulliparous singleton births with a gestational age ≥ 37 weeks. Cases with birthweights outside of the probable range for gestational age [18] were excluded from the study. Informed consent was not obtained since the study was based on de-identified data.

### Statistical analysis

Frequencies and percentages of the outcome variable and confounders were calculated separately by race and ethnic categories (non-Hispanic (NH) White, Black-NH, Hispanic, Asian-NH, or Native American-NH). The dichotomous outcome variable indicated whether women had a caesarean section for her first delivery. The choice of confounding variables was guided by a directed acyclic graph [19] and the variables available in the birth record files. These confounders included mother’s age (< 20, 20–24, 25–29, 30–34, 35–39, ≥40 years); mother’s marital status (married, unmarried); urban/rural designation of mother’s county of residence (urban, rural); mother’s education (< 12 years, high school degree, some college, at least a college degree); insurance status at the time of birth (Medicaid, private, self-pay, other); pre-pregnancy body mass index (BMI; kg/m^2^), defined as underweight (< 18.5), normal weight (18.5–24), overweight (25–29), obese (30–39), morbidly obese (40–49), or super obese (≥50); and tobacco use during pregnancy (yes, no). Unadjusted and adjusted risk ratios (RR) for having a caesarean section were calculated for different race/ethnicity categories with White-NHs as the reference using Poisson regressions with robust error variances [20, 21].

**Fig. 1:**
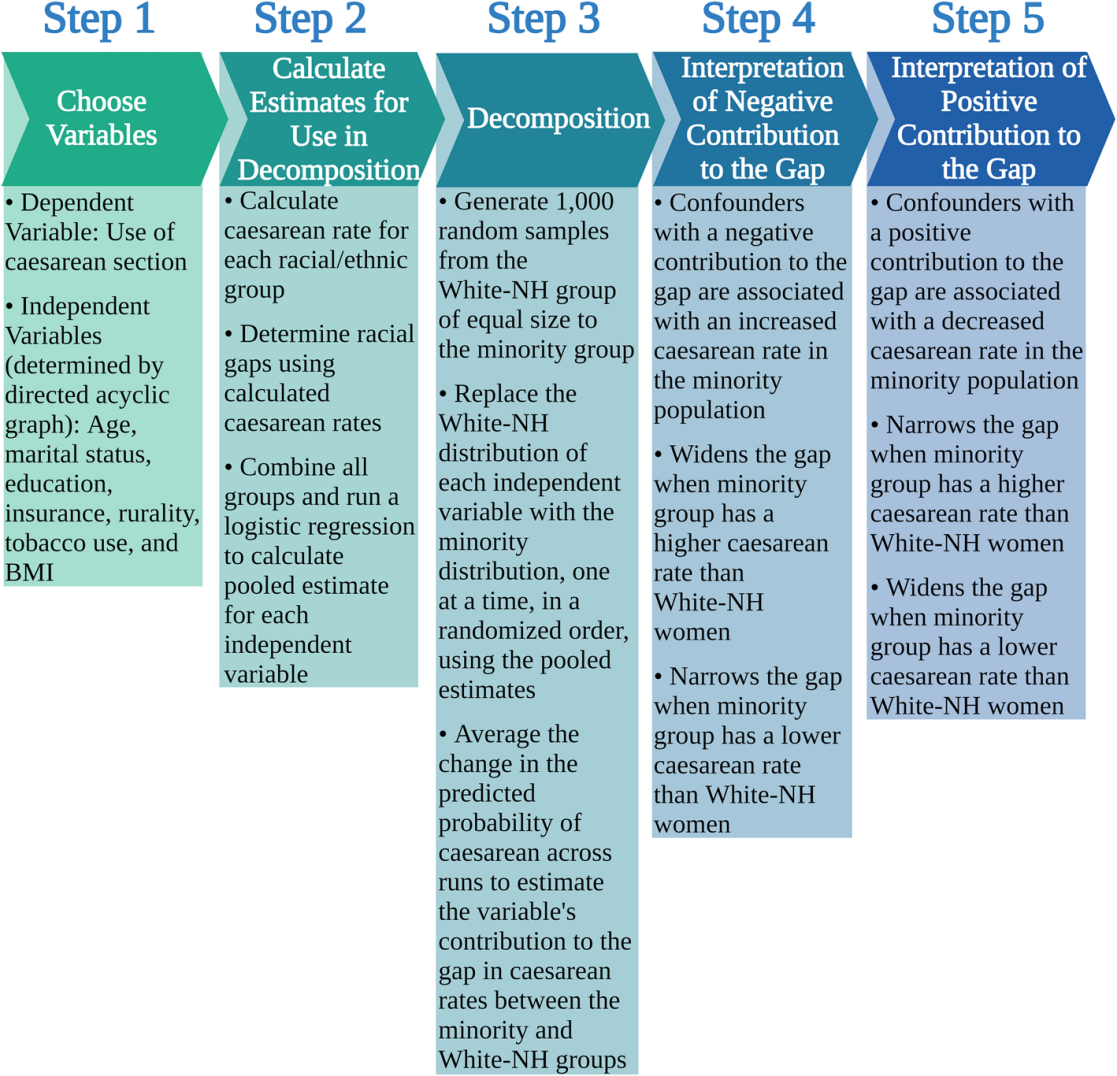
Steps in preforming and interpreting a Fairlie decomposition analysis. Created with BioRender.com

We then undertook a Fairlie decomposition technique specifically tailored for data with a dichotomous outcome [22]. The Fairlie decomposition technique has been previously used in a variety of settings to quantify and rank the contribution of independent variables based on their relevance in explaining observed differences between groups [23–28]. First, all racial and ethnic groups in the study were combined in order to obtain the pooled logistic regression coefficient estimates to be used in the decomposition analyses. Then, separately for each minority group, a random sample of White-NH cases of equal size to the minority sample size was chosen and the distribution of the minority replaced the White-NH distribution sequentially for each independent variable in order to calculate each independent variable’s contribution to the racial difference or gap in probability of the outcome; the order of variables was randomized, as outlined by Fairlie [29]. Finally, the process of randomly sampling a subsample of White-NH cases using a probability proportional to the size of the minority sample, with replacement, as well as a random ordering of the variables in the model to account for path dependence was repeated 1000 times to calculate the decomposition estimates, after which they were averaged across all runs. Contributions to the gap were given as probability estimates with associated 95% confidence intervals (CI) (Fig. 1).

## Results

The final sample for the race-only risk ratio model was 87,908, and the total sample for the full risk ratio model was 85,332 with an overall caesarean rate of 27.5%. The demographic characteristic of the population by race and ethnicity are shown in Table 1. The sample was primarily White-NH, followed by Hispanic and Black-NH. The highest rate of caesarean deliveries was seen in Asian-NH women, followed by Black-NH women, with the lowest rate of caesarean seen in Native American-NH women. With regards to BMI, more Black-NH women fell into the obese categories than White-NH women or Hispanic women, and Asian-NH women had the lowest rate of obesity. Native American-NH women had the highest rate of smoking during pregnancy, and Asian-NH women had the lowest rates.

**Table 1:** Characteristics of nulliparous women delivering at ≥37 weeks gestation by race/ethnicity

	Race/Ethnicity									
	White-NH		Black-NH		Asian-NH		Hispanic		Native American-NH	
	N	%	N	%	N	%	N	%	N	%
**Cesarean delivery**	19,013	27.8	1427	29.0	821	30.7	2650	24.7	264	23.4
**Maternal age (years)**										
< 20	8268	12.1	1697	34.4	146	5.5	3778	35.2	513	45.5
20–24	21,523	31.5	1893	38.4	513	19.2	4093	38.1	458	40.6
25–29	24,435	35.7	860	17.5	1077	40.2	1815	16.9	100	8.9
30–34	10,842	15.8	356	7.2	682	25.5	744	6.9	43	3.8
35–39	2837	4.1	105	2.1	212	7.9	264	2.5	13	1.2
≥ 40	521	0.8	17	0.3	48	1.8	53	0.5	1	0.1
**Married Marital Status**	43,425	63.5	1011	20.5	2224	83.0	3864	36.0	164	14.5
**Urban County of Residence**	37,553	54.9	4492	91.2	2324	86.8	5707	53.1	330	29.3
**Education**										
< 12 Years	4653	6.8	1265	25.7	294	11.0	4384	40.8	420	37.2
High School Degree	11,880	17.4	1573	31.9	393	14.7	3170	29.5	372	33.0
Some College	24,920	36.4	1595	32.4	492	18.4	2270	21.1	264	23.4
College Degree or Higher	26,863	39.3	493	10.0	1498	55.9	919	8.6	52	4.6
**Insurance Status at Time of Birth**										
Medicaid	17,072	24.9	3322	67.4	586	21.9	6418	59.7	755	66.9
Private	45,380	66.3	1195	24.2	1805	67.4	2807	26.1	166	14.7
Self-Pay	1647	2.4	168	3.4	138	5.2	966	9.0	67	5.9
Other	4327	6.3	243	4.9	149	5.6	557	5.2	140	12.4
**Pre-Pregnancy BMI**										
Underweight	2545	3.7	238	4.8	352	13.1	509	4.7	45	4.0
Normal Weight	36,704	53.6	2269	46.0	1769	66.1	5632	52.4	507	44.9
Overweight	15,917	23.3	1215	24.7	380	14.2	2581	24.0	238	21.1
Obese	10,413	15.2	917	18.6	137	5.1	1500	14.0	216	19.1
Morbidly Obese	1870	2.7	205	4.2	14	0.5	237	2.2	38	3.4
Super Obese	204	0.3	40	0.8	–	0.0	27	0.3	6	0.5
**Used Tobacco During Pregnancy**	10,337	15.1	454	9.2	69	2.6	527	4.9	273	24.2

Caesarean delivery rates were 27.8, 29.0, 24.7, 30.7, and 23.4% for White-NH, Black-NH, Hispanic, Asian-NH, and Native American-NH women, respectively. Table 2 shows the associations between confounders and the unadjusted (uRR) and adjusted (aRR) risk ratios of primary caesarean by race/ethnicity. Compared to White-NH women, both Hispanic (uRR = 0.88, 95% CI 0.85, 0.92) and Native American-NH (uRR = 0.86, 95% CI 0.77, 0.96) women had a lower risk of caesarean before adjustment while Asian-NH (uRR = 1.10, 95% CI 1.04, 1.17) women showed an increased risk of caesarean. After adjustment for all confounders listed in the Methods, Asian-NH (aRR = 1.21, 95% CI 1.14, 1.28) and Black-NH (aRR = 1.13, 95% CI 1.08, 1.19) women had an increased risk of caesarean relative to White-NH women; there was no longer any evidence of a difference in risk of caesarean between Hispanic (aRR = 0.99, 95% CI 0.95, 1.03) or Native American-NH (aRR = 0.93, 95% CI 0.84, 1.04) women relative to White-NH women.

**Table 2:** Unadjusted and adjusted risk ratios for cesarean delivery

	Unadjusted RR (95% CI)	Adjusted RR (95% CI)
**Race/Ethnicity**		
White - NH	1.00 (Reference)	1.00 (Reference)
Black - NH	1.04 (0.99, 1.09)	1.13 (1.08, 1.19)
Asian - NH	1.10 (1.04, 1.17)	1.21 (1.14, 1.28)
Hispanic	0.88 (0.85, 0.92)	0.99 (0.95, 1.03)
Native American - NH	0.86 (0.77, 0.96)	0.93 (0.84, 1.04)
**Maternal Age (years)**		
< 20	–	0.81 (0.77, 0.84)
20–24	–	1.00 (Reference)
25–29	–	1.21 (1.17, 1.24)
30–34	–	1.51 (1.45, 1.56)
35–39	–	1.94 (1.85, 2.02)
≥ 40	–	2.31 (2.14, 2.48)
**Married Marital Status**	–	0.98 (0.95, 1.01)
**Urban County of Residence**	–	0.94 (0.91, 0.96)
**Education**		
< 12	–	1.13 (1.07, 1.19)
High School Degree	–	1.13 (1.09, 1.18)
Some College	–	1.05 (1.02, 1.08)
At least College Degree	–	1.00 (Reference)
**Insurance Status at Time of Birth**		
Private	–	1.00 (Reference)
Medicaid	–	0.98 (0.95, 1.01)
Self-Pay	–	0.89 (0.83, 0.95)
Other	–	1.02 (0.96, 1.07)
**Pre-Pregnancy BMI**		
Underweight (<18.5)	–	0.82 (0.76, 0.88)
Normal (18.5–24.99)	–	1.00 (Reference)
Overweight (25–29.99)	–	1.34 (1.30, 1.38)
Obese (30–39.99)	–	1.76 (1.71, 1.81)
Morbidly Obese (40–49.99)	–	2.22 (2.13, 2.31)
Super Obese (≥50)	–	2.88 (2.67, 3.11)
**Used Tobacco During Pregnancy**	**–**	0.93 (0.90, 0.96)

Relative to women with normal pre-pregnancy BMI, the risk of caesarean increased as BMI increased. The risk of caesarean section was 34% higher for overweight women compared to normal weight women (aRR = 1.34, 95% CI 1.30, 1.38). For super obese women, risk of caesarean section was 188% higher compared to normal weight women (aRR = 2.88, 95% CI 2.67, 3.11). Similarly, the risk of caesarean increased with older maternal age while risk decreased with a higher level of maternal education. Additional factors significantly associated with lower risks of caesarean included urban residence, self-pay insurance, and tobacco use during pregnancy (Table 2).

Table 3 reports the results of the Fairlie analyses showing the relative contribution of various factors in explaining differences in use of caesarean. Variables with a positive percentage indicate protective factors associated with lower risk of caesarean among minority women relative to White-NH women, while variables with a negative percentage indicate risk factors associated with higher risk of caesarean among minority women relative to White-NH women.

**Table 3:** Factors identified by nonlinear decomposition analysis that contributed to the racial gaps in the caesarean rates

	Black - NH	Asian - NH	Hispanic	Native American - NH
	%	%	%	%
**Caesarean Rate Among White Women**	27.7	27.7	27.7	27.7
**Caesarean Rate Among Minority Group**	28.9	30.6	24.5	23.7
**Gap in Caesarean Rate**	−1.2	−2.9	3.2	4.0
**Estimated Effects of Explanatory Variable**^**a**^	**% (95% CI)**	**% (95% CI)**	**% (95% CI)**	**% (95% CI)**
**Maternal Age**	3.6 (3.4 to 3.9)	−2.8 (−2.9 to −2.6)	3.5 (3.3 to 3.8)	5.2 (4.8 to 5.6)
**Marital Status**	−0.2 (−0.6 to 0.1)	0.1 (0 to 0.3)	−0.2 (−0.4 to 0.1)	−0.3 (−0.7 to 0.1)
**County of Residence**	0.7 (0.4 to 0.9)	0.6 (0.4 to 0.8)	0.0 (0.0 to 0.0)	−0.4 (− 0.6 to − 0.3)
**Education**	−1.1 (− 1.4 to − 0.7)	0.2 (0.1 to 0.4)	−1.3 (− 1.8 to − 0.9)	−1.4 (− 1.9 to − 0.9)
**Insurance Status at Time of Birth**	0.3 (0.0 to 0.7)	0.1 (0 to 0.1)	0.5 (0.1 to 0.8)	0.4 (0.0 to 0.9)
**Pre-Pregnancy BMI**	−1.2 (− 1.3 to − 1.2)	3.7 (3.5 to 3.9)	0.3 (0.3 to 0.3)	−1.2 (− 1.3 to − 1.1)
**Tobacco Use During Pregnancy**	0.1 (0.1 to 0.2)	0.2 (0.1 to 0.4)	0.2 (0.1 to 0.3)	−0.2 (− 0.3 to − 0.1)

^a^Measures the magnitude and direction of contribution to the gap in caesarean rate for each explanatory variable. Variables with a positive estimated effect contribute to a lower caesarean section rate for the minority racial/ethnic group compared to the White group. Variables with a negative estimated effect contribute to a higher caesarean rate for the minority racial/ethnic group compared to the White group

For Black-NH women, who have 1.2% more caesareans than White-NH women (28.9% versus 27.7%), the decomposition analysis showed that maternal age, education, and BMI were the explanatory variables that contributed the most to explaining differences in caesarean rates (Table 3). Younger maternal ages of Black-NH women helped lower their caesarean rate relative to White-NH women and narrowed the gap by 3.6 percentage points (p.p.; 95% CI 2.4, 3.9). Conversely, lower levels of education (− 1.1 p.p., 95% CI -1.4, − 0.7) and higher pre-pregnancy BMI (− 1.2 p.p., 95% CI -1.3, − 1.2) among Black-NH women contributed to the higher caesarean rate in Black-NH women relative to White-NH women (i.e. widened the gap,).

Asian-NH women had 2.9% more caesareans compared to White-NH women (30.6% versus 27.7%). BMI and maternal age were the most determinant factors in the magnitude of the gap between the two groups (Table 3). The protective effect of BMI in Asian women reflects a higher proportion of normal or underweight BMI and much lower proportion of overweight or obese BMI in Asian-NH women compared to White-NH women (i.e. narrowed the gap, 3.7 p.p., 95% CI 3.5, 3.9). However, the relatively older maternal age contributed to the higher caesarean rates among Asian-NH women relative to White-NH women (i.e. widened the gap, − 2.8 p.p., 95% CI -2.9, − 2.6).

Native American-NH women had a 4.0% lower rate of caesarean when compared to White-NH women (23.7% versus 27.7%) and Hispanic women had 3.2% lower rates of caesarean compared to White-NH women (24.5% versus 27.7%). Since these two groups have *lower* rates of caesarean than White-NH, widening the gap in this instance refers to the minority having even lower caesarean rates than White-NH, and narrowing the gap refers to the minority having higher caesarean rates that are closer to the White-NH rate. Both Hispanic (3.5 p.p., 95% CI 3.3, 3.8) and Native American-NH (5.2 p.p., 95% CI 4.8, 5.6) women tended to have younger maternal ages than White-NH women, which contributed the most to their lower caesarean rates relative to White-NH women (i.e. widened the gap; Table 3). However, both Hispanic (− 1.3 p.p., 95% CI -1.8, − 0.9) and Native American-NH (− 1.4 p.p., 95% CI -1.9, − 0.9) women tended to have a lower level of education than White-NH women and Native American-NH women had a higher BMI (− 1.2 p.p., 95% CI -1.3, − 1.1) than White-NH women. Both of these factors narrowed the gap in caesarean rates between Hispanic or Native American-NH women and White-NH women.

## Discussion

Based on analysis of birth certificate data from a large, population-based cohort of singleton births, we report several important findings. First, the overall caesarean rate of 27.5% for women in our study is above the 24.7% goal for low-risk nulliparous pregnancies set by the Office of Disease Prevention and Health Promotion as part of the Healthy People 2020 initiative [7]. Additionally, the risk for caesarean was not distributed evenly across racial and ethnic groups, with Asian-NH (aRR 1.21, 95% CI 1.14, 1.28) and Black-NH (aRR 1.13, 95% CI 1.08, 1.19) women bearing an increased burden of caesareans compared to White-NH women. Our findings are similar to other studies that have examined the association between race/ethnicity and risk for caesarean across the United States [10, 11, 30–32]. A study of low-risk nulliparous women delivering at the University of Massachusetts Memorial Medical Center between 2006 and 2011 found that compared to White women, Asian women had 1.49 (95% CI 1.02, 2.17) time higher odds of delivering via caesarean and Black women had 1.43 (95% CI 1.07, 1.91) times higher odds [10]. At Kaiser Permanente Northern California, low-risk nulliparous Asian women had 1.59 (95% CI: 1.45, 2.06) times higher odds of delivering via caesarean and Black women had 1.73 times higher odds (95% CI 1.45, 2.06) compared to White women [11].

Despite the well-documented disparities in caesarean section rates based on maternal race/ethnicity, race/ethnicity itself is not a biological determinant of health [33, 34]. Instead, maternal race/ethnicity is crudely associated with socioeconomic injustices which drive the underlying biological and social causes of health disparities. This novel study attempts to elucidate some of these underlying causes for racial/ethnic disparities in caesarean section rates using a Fairlie decomposition analysis.

We found that among all the factors considered, maternal pre-pregnancy BMI, educational status, and age contributed the most to the observed differences in caesarean rates between minority women and White-NH women. BMI is a well-established risk factor for caesarean section, although less is known about the contribution of maternal BMI to racial disparities in caesarean rates. Linear trends have been established in several studies, with the leanest mothers having the lowest rates of caesarean deliveries [35–37], regardless of adjustments and stratification for possible confounders. The mean BMI in the United States has steadily increased over the past several decades [38], implying that women will enter pregnancy at higher weights. However, Black women bear a larger burden of obesity than other racial/ethnic groups [38]. Racial disparities in weight status are caused by a variety of systemic barriers to maintaining a healthy weight including but not limited to restricted access to fresh produce [39], targeted advertisements of unhealthy foods [40], and limited access to safe environments for exercising [41, 42]. Initiatives such as the Sisters Together: Move More, Eat Better Program [43] and Eat Smart, Move More North Carolina [44] attempt to address some of these systemic barriers, but further investment is needed to provide wide-spread access to weight-management resources. Overweight and obese women are also more likely to gain excessive weight during gestation, and less likely to lose excess weight after delivery [45]. This combination of high BMI at the onset of pregnancy and excessive weight gain during pregnancy place Black women at risk for caesarean deliveries, and for repeat caesarean deliveries in subsequent pregnancies if excess weight is not lost.

Our study identified maternal education as an additional variable which contributed to racial/ethnic disparities in caesarean rates among nulliparous women delivering at term. Lower education status has been previously associated with maternal preference for caesarean delivery in a cohort of Chinese mothers [46] and higher caesarean rates in a cohort of Belgian mothers [47]. Other studies indicate that maternal education may play a nuanced role in explaining caesarean rates. Kottwitz [48] demonstrated that lower maternal education was associated with higher caesarean usage for women with low access to obstetric care, but not for women with high access to obstetric care. Additionally, Castiglioni and Schmiedeberg [49] found that maternal education was intertwined with maternal age, with the effects of maternal education on caesarean rate becoming stronger after controlling for age. Effective, evidence-based initiatives to increase educational attainment for women struggling academically do exist, although more investment is needed at the federal, state, and community level to increase program access [50]. Examples of national educational initiatives which have the potential to increase high school completion rates for women prior to pregnancy include Talent Search (college-oriented programming) [51], Job Corps (vocational training, room, board, and medical care) [52], and Communities in Schools (academic, emotional, and nutritional support) [53].

Increasing maternal age has also been previously identified as a risk factor for caesarean delivery [30, 54–56]. Conversely, young maternal age (< 20 years old) is associated with other potentially severe morbidities including increased risk for maternal depression, anemia, preterm birth, and low birth weight [57]. Our results showed that nulliparous Asian-NH women tended to have older maternal ages (9.7% greater than 34 years old), while Black-NH, Native American-NH, and Hispanic women had younger maternal ages (34.4, 35.2, and 45.5% less than 20 years old, respectively). Schummers et al. [56] reported a similar distribution of maternal ages among a sample of over 16 million nulliparous women who gave birth in the United States. Maternal age is shaped by a complex network of economic, social, and personal factors which may be difficult to modify. However, overall birth rates for adolescents (15–19 years old), including Black-NH adolescents, have steadily declined since 2009 [8], due in part to public health initiatives such as the Title X program which provides low-cost family planning services [58]. The resulting upward trend in maternal age for Black-NH women [8], although necessary to prevent morbidities associated with adolescent pregnancy, may have the unintended consequence of widening the gap in caesarean rates between Black-NH and White-NH women. Further investment into initiatives that increase maternal educational level and/or decrease maternal obesity are urgently needed to minimize the potential negative impact of increasing maternal age on caesarean section rates for Black-NH women.

The strengths of this study include diversity in the profile of subjects and represents many types of institutions, including both rural and urban hospitals, private hospitals, and academic medical centers. Additionally, our results are highly relevant for practice and policy aimed towards reducing avoidable caesarean use in high-utilization groups, as we highlight three underlying factors that contribute to racial/ethnic disparities in caesarean sections.

Several limitations of this study deserve consideration. This study was based on routinely collected administrative data and is limited by the quality of available data. The data were collected in Nebraska and thus may not be generalizable to populations with different regional characteristics and clinical practice patterns; however, urban, suburban and rural populations are represented, and the racial and ethnic diversity demonstrated in the population is similar to midwestern populations in general [59]. Additionally, although maternal educational status, age, and BMI were associated with risk of caesareans, variables not included in Nebraska birth records also contribute to observed differences in caesarean rate. We do include information on maternal education and payer type in our analysis, which may serve as surrogate markers for socioeconomic status, and the sample included a mix of public and private pay, and a range of maternal education levels. Other important variables were not available for analysis, such as detailed information on facility of birth, maternal health conditions, birth position, reason for caesarean, anomalies at delivery, structural policies which influence maternal-fetal health outcomes, availability of community resources that promote health behaviors, access to obstetric care, food security, and household income. Future studies should attempt to quantify the effect of these variables on caesarean section rates.

## Conclusions

There are many possible causes of racial disparities in caesarean delivery rates, including individual, institutional, and structural level behaviors and policies. We found that demographic and social factors explain some of the differences in caesarean delivery rates across racial and ethnic groups. Given the contributions of maternal BMI, age, and educational status to racial disparities in caesarean deliveries, these findings provide clinicians and public health professionals targets for interventions to reduce unnecessary caesareans among minority women. Specific policy interventions which may contribute to decreasing caesarean rates include expanding high school completion programs, improving access to fresh fruits and vegetables through nutritional assistance programs, and providing spaces for physical activity in all communities. Future studies should investigate how other social, systemic, and facility level factors contribute to racial and ethnic disparities in caesarean section rates.

## Data Availability

The dataset analyzed during the current study was obtained from the vital records electronic registration system at the Nebraska DHHS. Data is available by contacting the Nebraska DHHS: https://dhhs.ne.gov/Pages/vital-records.aspx#birthcertificate

## Abbreviations

aRR: Adjusted Risk Ratio
BMI: Body Mass Index
CI: Confidence Interval
DHHS: Department of Health and Human Services
NH: Non-Hispanic
RR: Risk Ratio
uRR: Unadjusted Risk Ratio
